# The GPIIIA PlA2 polymorphism is associated with an increased risk of cardiovascular adverse events

**DOI:** 10.1186/1471-2261-10-41

**Published:** 2010-09-16

**Authors:** Gennaro Galasso, Gaetano Santulli, Federico Piscione, Roberta De Rosa, Valentina Trimarco, Raffaele Piccolo, Salvatore Cassese, Guido Iaccarino, Bruno Trimarco, Massimo Chiariello

**Affiliations:** 1 Department of Clinical Medicine, Cardiovascular and Immunologic Sciences, Federico II University School of Medicine, Naples, Italy

## Abstract

**Background:**

The clinical impact of PlA2 polymorphism has been investigated in several diseases, but the definition of its specific role on thrombotic cardiovascular complications has been challenging. We aimed to explore the effect of PlA2 polymorphism on outcome in patients with atherosclerosis.

**Methods:**

We studied 400 consecutive patients with coronary artery disease (CAD) undergoing percutaneous coronary intervention. A replication study was conducted in 74 hypertensive patients with cerebrovascular events while a group of 100 healthy subjects was included as control population. PlA genotype was determined by PCR-RFLP on genomic DNA from peripheral blood cells. Major adverse cardiac events (MACE), were considered as end points, and recorded at a mean follow up of 24 ± 4.3 months.

**Results:**

The frequencies of PlA2 polymorphism was similar between groups and genotype distribution was in Hardy-Weinberg equilibrium. In patients with CAD, the presence of PlA2 allele was associated with higher incidence of cardiac death (13.1% vs. 1.5%, p = 0.0001), myocardial infarction (10.7% vs. 2.6%, p = 0.004) and needs of new revascularization (34.8% vs. 17.7%, p = 0.010). Accordingly, the Kaplan-Meier analysis for event free survival in patients harboring the PlA2 allele showed worse long-term outcome for these patients (p = 0.015). Cox regression analysis identified the presence of PlA2 as an independent predictor of cardiac death (OR: 9.594, 95% CI: 2.6 to 35.3, p = 0.002) and overall MACE (OR: 1.829, 95% CI: 1.054 to 3.176, p = 0.032). In the replication study, the PlA2 polymorphism increased the risk of stroke (OR: 4.1, 95% CI: 1.63-12.4, p = 0.02) over TIA and was identified as an independent risk factor for stroke (B:-1.39; Wald: 7.15; p = 0.001).

**Conclusions:**

Our study demonstrates that in patients with severe atherosclerosis the presence of PlA2 allele is associated with thrombotic cardiovascular complications.

## Background

Atherosclerosis manifesting as myocardial infarction, angina pectoris, cerebral ischemia and peripheral artery disease is a multifactorial disease with the environment and genetics contributing to its pathogenesis. During the last decade several genes involved in the atherosclerotic process and their polymorphisms have been suspected to increase the thrombotic predisposition and to influence the risk for acute coronary syndromes. Among these genes, polymorphisms of those involved in platelet function have been extensively studied. Indeed, platelets play a pivotal role in atherothrombosis [1] and their function is strongly related to the interactions of the glycoprotein IIb/IIIa receptor (GP IIb/IIIa) and the von Willebrand factor, as well as fibrinogen, leading to platelets aggregation[2-4]. GP IIIa is a high polymorphic protein with platelet antigen 1 (PlA1) and 2 (PlA2) as the most common allelic isoforms [5]. In the PlA2 allele, cytosine is substituted for thymidine in exon 2, which is phenotypically translated in the substitution of proline for leucine at position 33 of the mature GP IIIa [6]. A previous in vitro study demonstrates that the PlA2 variant enhances the binding of the GPIIb/IIIa receptor to fibrinogen and therefore increases the platelet aggregation induced by agonists [7]. The clinical impact of PlA2 polymorphism has been investigated in several diseases, in which thrombus formation is a key pathogenetic factor, but the definition of the specific role of such polymorphisms on thrombotic coronary and cerebrovascular complications has been challenging. Weiss et al. [8] observed a strong association between the PlA2 polymorphism of the GP IIIa gene and acute coronary thrombosis, and this association was strongest in patients who had had coronary events before the age of 60 years, suggesting this polymorphism as an inherited risk factor for coronary thrombosis. These findings were further expanded on peripheral artery disease by Mikkelson [9] who reported an association between PlA2 variant and the progression of atherosclerosis in the abdominal aorta. Similarly, in the Copenhagen City Heart Study, a prospective study with 9,149 subjects, there was a three-fold and four-fold risk of ischemic cardiovascular disease and MI in men <40 years homozygous for PlA2 polymorphism [10]. On the other hand, several studies failed to confirm this association; indeed a metanalysis of 23 of such negative studies, showed the lack of association between the PlA2 allele and the risk of myocardial infarction and this negative result persisted even after subgroup analyses [11]. Therefore, up to date, available data are hugely uncertain and still debated. Several issues common to epidemiologic risk factor studies can be accountable for the difficulty encountered in reproducing the results of genetic association studies. Among these limitations there is inaccurate phenotyping [12]. In particular, atherosclerotic disease may present with different clinical manifestations. It is therefore pivotal to accurately select the clinical phenotype that can be affected by the genetic variability. To address this issue, and therefore gain more inside on the role of PlA2 polymorphism on atherothrombotic disease, we performed a prospective study in a cohort of patients selected for angiography documented severe coronary artery disease (CAD), which needed percutaneous coronary intervention (PCI). Moreover, to assess the role of this polymorphism on cerebrovascular disease, a replication study was performed in an independent population of hypertensive subjects, screened for large vessel atherosclerotic disease, and previous ischemic cerebrovascular events, namely stroke or transient ischemic attack (TIA). Finally, a group of 100 healthy subjects was included as control population of our study.

## Methods

### Patients

To assess the role of PlA2 polymorphism on cardiovascular disease we analyzed the incidence of this gene variant in a total of 574 unrelated individuals. A first group was constituted by 400 consecutive patients (mean age 60.5 ± 10, 83% male) undergoing elective or urgent PCI for CAD documented by a positive stress test or by Tl single photon emission computed tomography. To perform a replication analysis we selected an independent population of patients with cerebrovascular disease. Therefore, a population of 74 unrelated patients (mean age 61.58 ± 2, 63% male) with cerebrovascular events was examinated, recruited from those admitted to the Hypertension Diagnosis and Care Outpatient Clinic of "Federico II" University of Naples and participating in the 'Campania Salute Project'[13]. Finally, to identify normal distribution of the PlA genotype, we enrolled a control population of 100 gender- and age-matched healthy unrelated individuals (60 ± 10 years, 83% male), recruited from blood donors of our blood bank. Controls were free from heart disease, medication use, and cardiovascular risk factors, except for smoking habits. To help to diminish the likelihood of bias and reduce population stratification case and controls individuals were drawn from the same geographic region (Campania, Southern Italy) and matched for age, sex and race. All clinical data and biochemical features of enrolled patients were stored in a computerized database. A written informed consent was obtained from all patients according to the Ethics Committee of the "Federico II" University of Naples School of Medicine. The Ethic Committee regulations of our Institution approved the study protocol.

### Percutaneous coronary intervention

PCI was performed according to the American Heart Association/American College of Cardiology guidelines [14]. Antegrade perfusion was graded by Thrombolysis In Myocardial Infarction (TIMI) criteria [15]. Angiographic lesion morphology was classified according to American Heart Association/American College of Cardiology classification [16]. Stenoses >50% were considered significant. Bare-metal stents (BMS) were used in the 80% of patients, while drug-eluting stent (DES) in the remaining 20%. Glycoprotein IIb/IIIa inhibitors were used in 30% of patients. After the procedures, all patients were on dual antiplatelet therapy with aspirin and clopidogrel for at least 30 days after BMS implantation and for 12 months after DES. Other cardioactive drugs for long-term medical treatment were left to the discretion of the attending cardiologists.

### Outcome evaluation

In the evaluation of long-term clinical outcome, major adverse cardiac events (MACE) were considered as end points, including cardiac death, acute myocardial infarction (AMI), and needs of any new myocardial revascularization (considering re-PCI or coronary artery by-pass graft). All deaths were considered as cardiac, unless it was unequivocally proven non-cardiac. AMI was defined as recurrent chest pain with ST-segment or T-wave changes and recurrent elevation of cardiac enzyme levels. The follow-up was based on a direct systematic review of all patients' clinical files for a mean study period of 24 ± 4.3 months, contacting relatives or a patient's physician when necessary. Follow-up was completed in all patients and data stored in a computerized database. A second population was identified among the hypertensive cohort of patients afferent to the 'Campania Salute Project' [13]. We selected 74 patients affected by cerebrovascular events, either TIA or stroke. In particular, the diagnosis of TIA or stroke was confirmed by neurological evaluation according to current standards for care [17]. This included patient personal and family history, physical examination, computed tomography or magnetic resonance imaging, and laboratory testing. The diagnosis of stroke was based on WHO criteria, confirmed by TC or MR, when needed. The cause of stroke was identified in large-vessel disease, according to the clinical features and results of the diagnostic workup, based on TOAST pathophysiological classification [18]. TIA was defined as an episode of focal neurological symptoms with abrupt onset and rapid resolution lasting <24 hours that is due to altered circulation to a localized portion of the brain [19].

### GpIIIa genotyping

Using a commercially available kit (Midiprep DNA, Qiagen, Valencia, California), genomic deoxyribonucleic acid (DNA) was isolated from 2 ml of the peripheral blood samples. PlA2 polymorphism was studied using combined polymerase chain reaction (PCR) and restriction fragment length polymorphism technique (RFLP) on PCR amplified products using PCR conditions and primers on a Thermocycler (MJ Research, St. Bruno, Canada) and DNA polymerase (TAQ, Qiagen) as previously reported [20,21].

### Statistical Analysis

Continuous variables are presented as mean ± SD and categorical variables as absolute number and percentage value. Differences between groups were assessed using univariate analysis of variance for continuous variables, with a Bonferroni post-hoc test for evaluation of multiple comparisons. Categorical variables were analyzed by chi-square test, and odds ratio (OR) with 95% confidence intervals (CIs) or by the Fisher exact test when the expected values in any of the cells of the test, given the frequencies and the overall sample size, was below 10; p value <0.05 was considered significant. Difference in event-free survival between groups were evaluated by the Kaplan-Meier method, comparisons were made using log-rank test. A Cox regression analysis was conducted for the PlA2 polymorphism considering age, cardiovascular risk factors, medication use, left ventricular ejection fraction, angiographic characteristics, and interaction of the PlA2 polymorphism in both the study populations. The software used was SPSS 16 (SPSS Inc., Chicago, Illinois).

## Results

### Frequencies of PlA2 polymorphism in study population

As reported in Table 1, the frequencies of PlA2 polymorphism was similar between study and control group. The genotype distribution was in Hardy-Weinberg equilibrium. The allele frequency for PlA1 and PlA2 was, respectively, 84% and 16% in patients with CAD, 82.3% and 17.7% in hypertensive subjects with cerebrovascular events, and 84.5% and 15.5% in the control healthy group.

**Table 1:** Genotype distribution of PlA2 polymorphism in study populations

Genotype	CAD patients	Hypertensive patients	Control population	P
**PlA1/A1**	70.4	67.8	72	0.79
**PlA1/A2**	27	29.1	25	0.91
**PlA2/A2**	2.6	3.1	3	0.81

CAD: Coronary artery disease

### Clinical and angiographic characteristics in patients with coronary artery disease

Accordingly to the allelic distribution of the PlA2 allele we divided patients with CAD into 2 groups: group 1 (300 patients, PlA1/PlA1 genotype; 90% male, mean age 60 ± 10 years) and group 2 (100 patients, PlA1/PlA2 and PlA2/PlA2 genotypes; 81% male, mean age 62.1 ± 8.6 years). As reported in tables, no differences were noted between the two groups regarding baseline clinical (table 2) and angiographic (table 3) features.

**Table 2:** Clinical characteristics of CAD patients

Variable	PlA1 (n = 300)	PlA2 (n = 100)	p Value
**Age**	60 ± 10	62.1 ± 8.6	0.72
**Male sex (%)**	90	81	0.03
**Hypertension (%)**	56	57	0.82
**Diabetes (%)**	29.3	19.4	0.11
**Smoking (%)**	58.6	53.8	0.45
**Dyslipidemia (%)**	55.2	53.8	0.81
**Obesity (%)**	9.5	7.7	0.89
**Family history of CAD (%)**	38.2	33.4	0.37
**Previous AMI (%)**	10.9	15.4	0.35
**Previous PCI (%)**	10.9	15.4	0.35
**Previous CABG (%)**	9.1	3.8	0.15
**LVEF prior to PCI, mean ± SD**	47 ± 10	51 ± 8	0.06

PCI: Percutaneous coronary intervention

LVEF: Left ventricular ejection fraction

**Table 3:** Angiographic characteristics of CAD patients

Variable	PlA1 (n = 300)	PlA2 (n = 100)	p Value
**Coronary artery disease (%)**			
**LAD**	61.9	50	0.27
**Cx**	33.6	29	0.37
**Dx**	45	50	0.30
**Multivessel Disease (%)**	28	30	0.49
**Lesion type, B2/C (%)**	78	85	0.55
**BMS (%)**	74	66	0.56
**DES (%)**	26	34	0.57
**TIMI 3 post PCI (%)**	97.5	98	0.89
**cTFC post (mean ± SD)**	15.10 ± 6	14.25 ± 8	0.76
**Lesion length (mm)**	15.54 ± 6.59	16.48 ± 8.74	0.13
**Preprocedural stenosis (%)**	86.55 ± 10.19	85.05 ± 11.48	0.11
**Stent/patient (n)**	1.08 ± 0.32	1.07 ± 0.26	0.74

BMS: Bare metal stent, Cx: Left circumflex coronary artery, DES: Drug eluting stent, Dx: Right coronary artery, LAD: Left anterior descending coronary artery.

### Medication use

There were no differences between groups regarding medication use at the time of PCI and during follow-up time. In particular, at follow-up time both groups were similarly treated, using dual antiplatelet therapy (99% vs. 98% group 2, p = NS), calcium antagonist (19% vs. 18% group 2, p = NS), beta-blockers (45% vs. 47% group 2, p = NS), angiotensin-converting enzyme inhibitors (36% vs. 34% group 2, p = NS), and statins (42% vs. 40% group 2, p = NS).

### Long-term follow-up

**Figure 1:**
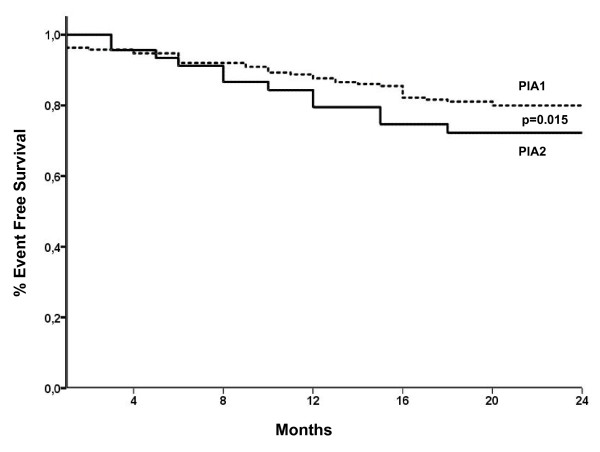
**Observed event free survival curve at follow up**: Patients with coronary artery disease harbouring the PlA2 allele of the GP III a gene (group 2) had a higher incidence of MACE with reduced event free follow up time as indicated by the Kaplan Meier analysis.

There were no significant differences between groups regarding MACE incidence during hospitalization. Interestingly, at long-term follow-up, the rates of MACE were significantly higher in PlA2 group compared to PlA1 (43.5% vs 25.8%, p 0.018). In particular, patients with PlA2 allele showed a significant increased rate of cardiac death (14.6% vs 5.3%, p = 0.025), AMI (12% vs 4.5%, p = 0.043) and new myocardial revascularization (34.8% vs 17.7%, p = 0.010). Accordingly, the Kaplan-Meier analysis for event free survival in patients harboring the PlA2 allele (Figure 1) showed worse long-term outcome for these patients (p = 0.015). Cox regression analysis identified the presence of PlA2 allele as an independent predictor for cardiac death (OR: 9.594, 95% CI: 2.6 to 35.3, p = 0.002) and overall MACE (OR: 1.973, 95% CI: 1.039 to 3.747, p = 0.036). There were no interactions noted for this relationship when considering age, sex, risk factors for CAD, multivessel disease, previous myocardial revascularization, number of treated lesions, and basal left ventricular ejection fraction.

### Effect of PlA2 polymorphism on cerebrovascular events in hypertensive patients

To analyze in a different population the impact of this polymorphism on atherothrombotic events we analyzed the impact of PlA2 in hypertensive patients with history of cerebrovascular events according to the level of cerebral damage (TIA or stroke). The characteristics of these patients are depicted in table 4. Both the TIA and stroke groups presented with an elevated cardiovascular risk due to the presence of many risk factors which distribution did not differ between the two groups. Interestingly, PlA2 allele was significantly more represented among patients with stroke than patients with TIA (stroke:46.4%; TIA:17.4%; p = 0.01). This polymorphism indeed increased the risk of stroke in this high risk population (OR: 4.1, 95% CI: 1.63-12.4, p = 0.02) over TIA. Finally, a multiple regression analysis corrected by common risk factors for cerebrovascular events, showed that PlA2 allele as an independent risk factor for stroke (B:-1.39; Wald: 7.15; p = 0.001).

**Table 4:** Characteristics of hypertensive patients

Variable	Hypertensives with TIA (n = 46)	Hypertensives with stroke (n = 28)	P value
**Age (years)**	62.06 ± 1.63	61.1 ± 2.60	0.77
**Male sex (%)**	54.3	71.4	0.15
**Diabetes (%)**	9.7	10.6	0.88
**Smoking (%)**	55.3	58.1	0.75
**Dyslipidemia (%)**	51.1	51.6	0.95
**SBP (mmHg)**	150.08 ± 3.23	147.5 ± 2.88	0.42
**DBP (mmHg)**	87.94 ± 1.78	88.83 ± 2.16	0.97
**LVMI (g/m**^**2**^**)**	127.09 ± 3.64	127.82 ± 2.68	0.98

DBP: Diastolic blood pressure

LVMI: Left Ventricle Mass Index

SBP: Systolic blood pressure

## Discussion

The main finding of this study is the relationship between the PlA2 gene variant and the incidence of major adverse cardiovascular events in patients harbouring the PlA2 gene polymorphism. Indeed, in patients with CAD undergoing PCI we found that the presence of PlA2 allele associated with a significantly worse prognosis with a higher incidence of cardiac death, AMI, and new myocardial revascularization. Moreover, in an independent population of hypertensive patients with large vessel atherosclerosis and previous cerebrovascular events, patients harbouring the PlA2 gene polymorphism presented with a significantly higher incidence of stroke over TIA. Taken together these data suggest an effect for the PlA2 gene polymorphism in the development of more severe thrombotic complications in high risk patients.

Since the original report from Weiss et al.[8] indicating PlA2 polymorphism as a risk factor for myocardial infarction or unstable angina, several studies have investigated this polymorphism in the effort to discover a novel thrombogenic risk factor but to date results have been inconclusive and often controversial [8,11,22]. The inconsistence of findings in literature can be mostly attributed to differences in the design as well as to the choice of the control group and the endpoint of the studies. Moreover, studies differ in the variation of environmental factors and ethnicity, present biases in the selection of patients and controls and often aim to different clinical endpoints. Since atherosclerosis is a multifactorial disease, it would be too simplistic to explain interindividual variations based on genetic inheritance alone. Indeed several challenges exist in identifying the genetic determinants of such a complex disease including genetic heterogeneity, gene-gene and gene-environment interactions[12]. Furthermore, interaction of multiple genes that are in linkage disequilibrium and simultaneous studies of several genes may reveal associations that at present seem to be weak. Nevertheless, such approaches are costly, and impose the use of large populations and heavy statistic modeling in order to carefully peruse small impact of single gene variants on multifactorial disease [12]. There is still room for candidate gene association study that can be performed in relatively small populations, that are carefully characterized and selected for homogeneity[12]. We also have recently underlined the need for accurately selection of patients in association studies in order to identify the populations in which single gene polymorphisms may be more determinant in complex phenotypes such as atherosclerosis [20,21]. Therefore, since platelets play a major role in the development of atherosclerotic thrombosis and acute ischemic events, we investigated the role of the PlA2 gene polymorphism in higher risk populations such as patients with severe CAD requiring mechanical revascularization and hypertensive patients with previous cerebrovascular events. Interestingly, in the CAD population, we observed that the presence of PlA2 patients was associated with a 3 times higher risk of death or 2.8 increased risk of AMI. Noteworthy, patients harbouring the PlA2 allele presented an higher risk of needing new revascularization at follow up with 50% of patients undergoing new PCI for the occurrence of a new myocardial infarction. This is consistent with data by Kastrati et al[23] reporting a higher risk of restenosis after coronary stent placement in these patients. Moreover, in an independent population of hypertensive patients with cerebrovascular events, the presence of the PlA2 allele was associated with a 4.1 higher risk to develop stroke rather than TIA. Thus, these data suggest that, in a high risk clinical scenario represented by patients with a greater prevalence of atherothrombotic risk factors, the presence of PlA2 is associated with a more aggressive disease leading to an higher incidence of major ischemic events and to an unfavourable outcome. Our results only apparently differ from the finding observed in a subgroup analysis of the Physicians' health study (PHS) [24] by Ridker et al.[25] which prospectively studied the risk associated with the PlA2 polymorphism for myocardial infarction, stroke or venous thromboses and reported no associations between PlA2 polymorphism and the relative risk to develop any cardiac or cerebro-vascular event. Indeed, as already remarked by previous studies [26,27], the PHS was conducted in a very healthy population, with an event rate 4 times less than general population, and therefore with a very low risk of cardiovascular events. Also, our analysis focused on the outcome and the incidence of MACE in patients that already had a clinical manifestation of the disease, while the PHS aimed to the occurrence of the first event.

Limitations of our study include those inherent to any prospective but observational study. Moreover, since this is an association study, we cannot rule out the presence of a possible linkage disequilibrium with other neighboring genes that might explain the significant association with atherosclerotic phenotype or adverse prognosis.

The study was conducted in patients with severe CAD undergoing PCI while the replication study was performed in hypertensive patients with previous cerebrovascular events; therefore, our findings need to be confirmed in further larger patient populations. Nevertheless, recent studies[20,21] underlines the need for selection of patients in association studies in order to identify the populations in which single gene polymorphisms may be more determinant in complex phenotypes such as CAD.

## Conclusions and clinical implications

Our study suggests that the PlA2 polymorphism is associated with a more aggressive atherothrombotic disease and adversely affects the prognosis in these high risk patients. Indeed, in patients with severe CAD undergoing PCI the presence of PlA2 allele is associated with increased risk of death, myocardial infarction and new myocardial revascularization. Moreover, high risk hypertension patients harbouring the PlA2 allele show an increased risk to develop severe cerebral damage. Therefore considering the atherothrombotic disease, the PlA2 genotype could be useful to physicians in targeting antithrombotic strategies and therapeutic options. Since atherosclerosis is a multifactorial disease, future studies on interactions between environmental factors, common cardiovascular risk factors and platelet associated genetic determinants are warranted.

All Authors substantially contributed to the production of the manuscript.

FP and GG identified the hypothesis of the study. GG, GS and GI drafted the manuscript. Furthermore, FP was in charge of the percutaneous revascularization procedures, together with GG in patients with coronary artery disease. RDR, RP, SC and VT carried out the molecular genetic studies, performed the statistical analysis, patients' data collection and follow up. MC and BT are respectively, the heads of the Division of Cardiology and Division of Internal Medicine, at our Institution, and they finally approved the submission of the manuscript.

All authors read and approved the final manuscript.

## Pre-publication history

The pre-publication history for this paper can be accessed here:

http://www.biomedcentral.com/1471-2261/10/41/prepub
